# IQOS Marketing in the US: The Need to Study the Impact of FDA Modified Exposure Authorization, Marketing Distribution Channels, and Potential Targeting of Consumers

**DOI:** 10.3390/ijerph181910551

**Published:** 2021-10-08

**Authors:** Carla J. Berg, Lorien C. Abroms, Hagai Levine, Katelyn F. Romm, Amal Khayat, Christina N. Wysota, Zongshuan Duan, Yael Bar-Zeev

**Affiliations:** 1 Milken Institute School of Public Health, George Washington University, Washington, DC 20052, USA; lorien@gwu.edu (L.C.A.); kromm@gwu.edu (K.F.R.); cwysota@gwu.edu (C.N.W.); zduan3@gwu.edu (Z.D.); 2 GW Cancer Center, George Washington University, Washington, DC 20052, USA; 3 Braun School of Public Health and Community Medicine, The Hebrew University of Jerusalem and Hadassah Medical Organization, Jerusalem 9112102, Israel; hlevine@hadassah.org.il (H.L.); amal.khayat@mail.huji.ac.il (A.K.); Yael.Bar-Zeev@mail.huji.ac.il (Y.B.-Z.)

**Keywords:** tobacco industry, tobacco control, marketing, heated tobacco products

## Abstract

IQOS, the leading heated tobacco product globally, recently received ‘reduced exposure’ authorization from the US Food and Drug Administration. Independent research focusing on IQOS marketing and potential impact on consumers’ perceptions and behavior, and ultimately public health, is critical. The literature to date has underscored several concerns. First, Philip Morris’s (PM’s) marketing distribution requires scrutiny, particularly given its innovative promotional strategies. For example, IQOS is distributed via unique points-of-sale (POS; e.g., specialty and pop-up stores, “corners” in convenience stores) and uses various other opportunities (e.g., social media, sponsored events, direct-to-consumer). Second, although PM claims that IQOS’ target market is current combustible tobacco users and not young people, the literature indicates that in some populations, IQOS use is equally prominent among smokers and nonsmokers, and that specific subgroups (e.g., young adults, women) are targeted. Third, the impact of IQOS’ use of ad content promoting IQOS health benefits must be studied (e.g., how consumers interpret modified exposure messages). In conclusion, surveillance of IQOS marketing, particularly following reduced exposure authorization, is critical for obtaining valuable data to estimate population impact, particularly among population subgroups (e.g., young adults), and inform future tobacco regulation. These considerations have implications beyond IQOS—to other products and companies.

## 1. Introduction

In order to stay in or enter the US market, manufacturers are required to submit a Premarket Tobacco Product Application (PMTA) to the Food and Drug Administration (FDA) for any new tobacco product (or modification of products) commercially marketed after February 2007, which also includes e-cigarettes [1]. Manufacturers can also apply for the Modified Risk Tobacco Product (MRTP) category, which, if approved, would grant authorization to use “reduced risk” or “reduced exposure” messaging in their marketing [2]. Among new tobacco products emerging and expanding globally [3,4,5,6] are heated tobacco products (HTPs; electronic tobacco products that heat tobacco), which have shown dramatic increases in use and consumer interest [7,8]. This paper focuses on the global HTP leader IQOS by Phillip Morris (PM) [9]; IQOS was first released in Japan in 2014 and is now sold in >60 countries and has >17 million users [10].

As a result of IQOS’ global prominence among the HTPs and the recent FDA regulatory activities related to IQOS, IQOS is a highly relevant and timely tobacco product as it relates to how the tobacco market in the US has evolved under new FDA regulatory activities. In April 2019, IQOS received FDA authorization to enter the US market. In July 2020, the FDA authorized IQOS to use in its marketing “reduced exposure” claims (i.e., reduced exposure to harmful substances relative to cigarettes) [2]—but did not authorize its use of “reduced risk” claims (i.e., safer or less harmful than cigarettes) [11]. During this time, IQOS began establishing its US market. IQOS opened its first US-based store in Atlanta, Georgia in September 2019. As of May 2021, IQOS is sold in four states: Georgia (Atlanta, Buford), Virginia (Richmond, Tysons), North Carolina (Charlotte, Raleigh), and South Carolina (Charleston, Myrtle Beach) [12]. While the use of IQOS in the US is currently low, interest and use are growing [5,13]. Moreover, globally, PM intends to expand its HTP portfolio. In 2021, PM has stated its goal is that half of its revenues in the next 4 years should be represented by smoke-free products, of which IQOS is a leading brand [14].

To inform regulatory decisions, the FDA needs data to estimate the impact of these products and related marketing on consumer behavior and ultimately public health, particularly as IQOS expands across the US. Various types of market research (e.g., market segmentation, discrete choice experiments) [15,16,17,18] inform the industry’s advertising content, promotional strategies, and distribution channels [19,20]. There is vast literature, particularly regarding combustible cigarettes, indicating the influence of advertising across marketing channels (e.g., online, via social media, at point-of-sale (POS)) on use behavior and perceptions among adults and youth [21,22,23,24,25,26,27]. While manufacturers’ applications to the FDA must include data to estimate product and marketing impact, independent research suggests that these findings often underestimate such impact [28,29,30,31,32,33,34,35,36], thus calling for ongoing comprehensive surveillance by independent researchers. This commentary aims to underscore high-priority research areas, which are identified based on research published primarily in the past 3 years regarding IQOS marketing and related regulatory changes and policy in the US.

## 2. IQOS Marketing Channels

The increasing restrictions on tobacco advertising via traditional channels globally has led to a shift in paid advertising for tobacco products to just a few channels – which in the US includes mostly print, online/mobile, and at POS [37,38]. A recent study found that paid advertising in the US during IQOS’ first 2 years (Aug 2019 to April 2021) totaled $4.9 million, with the vast majority spent on print ads (99%) but the greatest circulation via online/mobile (92%) [38]. The literature underscores that the online environment is a prominent venue for surveillance [38] and the need to monitor a broad range of non-traditional marketing channels [39,40,41,42].

**Figure 1:**
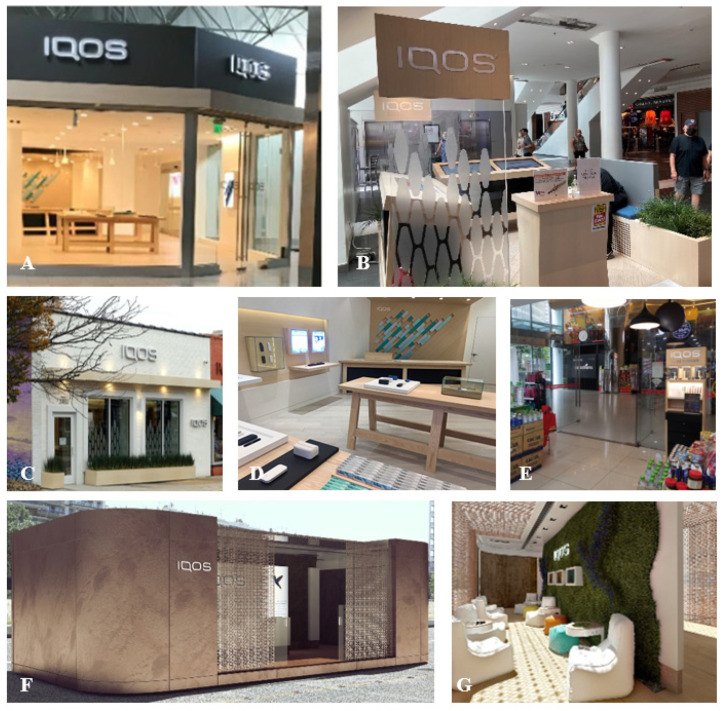
Types of IQOS points-of-sale. Notes: (**A**): Specialty Store, Lenox Mall, Atlanta, Georgia, US, Fall 2019 [37]; (**B**): Kiosk, Pentagon City Mall, Arlington, Virginia, US, Summer 2021 (photo by authors); (**C**,**D**): Specialty Store, Richmond, Virginia, US, Spring 2021 (photo by authors); (**E**): Corner, Kuala Lumpur, Malaysia, Fall 2019 [49]; (**F**,**G**): Pop-up, Thessaloniki, Greece, Summer 2018 [50].

One key marketing opportunity is the POS, given the widely adopted restrictions on various other marketing channels in many countries. IQOS and associated heatsticks (HEETS, which are the tobacco-containing sticks that are inserted into the IQOS device and then heated) are sold in unique retail settings, including their IQOS specialty stores, kiosks, pop-up stores, and IQOS corners of convenience stores (see Figure 1 for examples of retail settings), while HEETS are also sold at traditional retail outlets (e.g., pharmacies, convenience stores) [43]. Within these novel settings, they also employ unique promotional strategies [37,44,45,46,47]. For example, a Canadian study found that specialty stores use bold promotional activities, including deals involving exchanging cigarette packs or lighters for IQOS, social events, and membership programs [44]. Regarding traditional retailers, prior research in Israel [46,47] indicated that IQOS/HEETS were prominently displayed (often using large displays/stands) and often placed near candy/toys and within 1 m from the floor [45,46], and that PM directly incentivizes retailers based on their IQOS/HEETS sales and provides instructions regarding how to communicate with consumers about IQOS [48]. Research regarding IQOS emergence in the US [37] has indicated the various types of POS in Atlanta and promotional strategies used, such as low-cost personal trials, education provided by staff, and price promotions (e.g., “refer a friend”), among others [37]. These findings also highlight the need to examine direct-to-consumer marketing strategies that are much more difficult to capture using traditional surveillance approaches.

A special consideration is “marketing” via social media. Social media—particularly Twitter, Facebook, and Instagram [51]—are prominent communication channels of tobacco-related content (including IQOS) [42,52,53]. While social media companies generally prohibit paid tobacco marketing on their sites, the tobacco industry may have their own social media accounts and/or pages. At present, PM has a number of social media accounts for IQOS, for example, on Facebook (GetIQOS.US) and Instagram (@get_IQOS). The tobacco industry may also generate unpaid or seemingly ‘organic’ content to create a community of interested users, which then provides a vehicle for influencing consumers and providing links to external content (e.g., websites for product purchases) [52,54,55,56,57]. Indeed, IQOS historically has used social influencers engaging via social media in various markets globally [41,42]. A recent study of IQOS-related tweets in 2020 across sources, including individuals giving personal testimonies and IQOS vendors, documented that key themes included health claims and tweets commonly linked to news stories, which could be sources of further content that have been understudied [58]. Given social media’s reach and impact, examining sources of tobacco-related content, the content generated, and its reflection of industry and consumer behavior over time is warranted. This is particularly critical, given social media’s high potential to spread misleading information among consumers [59,60,61,62]. Notably, the FDA’s regulatory purview extends to paid tobacco marketing [63,64,65] but has not been clearly extended to unpaid promotion via social media; nonetheless, such promotion may influence consumers [27,52,54,55,56,57].

In short, the strategies used to promote IQOS via paid advertising warrant further research. In particular, the unique PM promotional strategies used at POS are especially intriguing and require a better understanding of how retail IQOS marketing practices differ from other tobacco products. Moreover, research is needed regarding marketing communications that are not explicitly paid or not clearly connected to the industry, such as via social media, special sponsored events, and direct-to-consumer.

## 3. IQOS’ Target Consumers

A critical component of marketing is identifying target markets for any product, which drives decisions regarding ad content and marketing distribution channels [66]. PM asserts that its target market for IQOS is current cigarette smokers and that they are specifically not targeting young people [2,67,68]. A first step in determining whether this is bearing out is to examine trends in use among those with differing tobacco use histories and among key sociodemographic groups. In the US, current smokers have shown greater HTP use [6,13]. Research in other countries (e.g., Italy [69], Korea [70]) indicates mixed findings—some showing that IQOS users are more likely to smoke conventional cigarettes and/or e-cigarettes [70] and other work showing that never smokers are equally or more likely than current smokers to have already tried or intend to try IQOS [69]. Further contradicting the stated target market, a sizeable proportion of US youth and young adults (38.6%) [5] are interested in IQOS, and relative to older adults, young adults are more likely to use HTPs [6]. In addition, men and racial/ethnic minorities may be more likely to use HTPs [6,13].

Prior literature indicates that in various global markets, young adults are targeted by IQOS marketing, which has used launch events staffed by young adults, sponsorships of concerts and art festivals, and using distribution channels with a young adult target market (e.g., via specific magazines/websites) [41,42]. Specific to the US, a study of IQOS marketing during its initial 2 years indicated targeting of young adults and women, given that young women were depicted in ads more frequently than men and that the number of campaigns and amount of ad spend was largely devoted to marketing channels with audiences predominantly under age 25 and/or women [38]. Marketing content and distribution channels also indicate targeting of those interested in technology and innovation, which may skew toward younger populations [38]. Moreover, in various global markets, the types of POS environments [37,41] and where they are located [71] indicate potential targeting of young people.

In short, the literature to date undermines PM’s claims regarding their target markets, particularly raising concern about the targeting of nonsmokers, especially young adults and women. Moreover, there are also data that underscore the need to assess subgroups historically targeted by the tobacco industry and who face tobacco-related health disparities (e.g., racial/ethnic minorities, sexual/gender minorities) [72,73].

## 4. IQOS’ Health-Related Ad Content 

Given FDA authorization to use “reduced exposure” messaging for IQOS, particularly worthy of examination are advertising messaging strategies that promote IQOS’ potential health benefits. As noted above, the FDA authorized IQOS’ use of “reduced exposure” messaging but not “reduced risk” [11]. Notably, some of the authorized language is concerning: (1) reduced exposure claims may be interpreted as reduced risk [74,75,76]; and (2) consumers often misinterpret messaging about “switching completely” from traditional cigarettes to IQOS [74,75,76]. Outside of the FDA MRTP references, IQOS ads often claim that IQOS (vs. cigarettes) is a “cleaner” product (i.e., “less ash”, “less odor”), poses “reduced risk” [77,78,79], is more acceptable to nonsmokers [37,79], and is a satisfactory alternative to cigarettes (e.g., “real tobacco”), despite mixed findings [33,34,79]. PM’s efforts to distinguish IQOS from vaping products are also noteworthy, which has been notable since e-cigarette and vaping-associated lung injuries (EVALI) [80]. Research in the US has documented a substantial number of IQOS ads featuring text addressing this: for example, “It’s not a vape. It’s not an e-cig. It’s real tobacco with less odor and no ash” [38].

**Figure 2:**
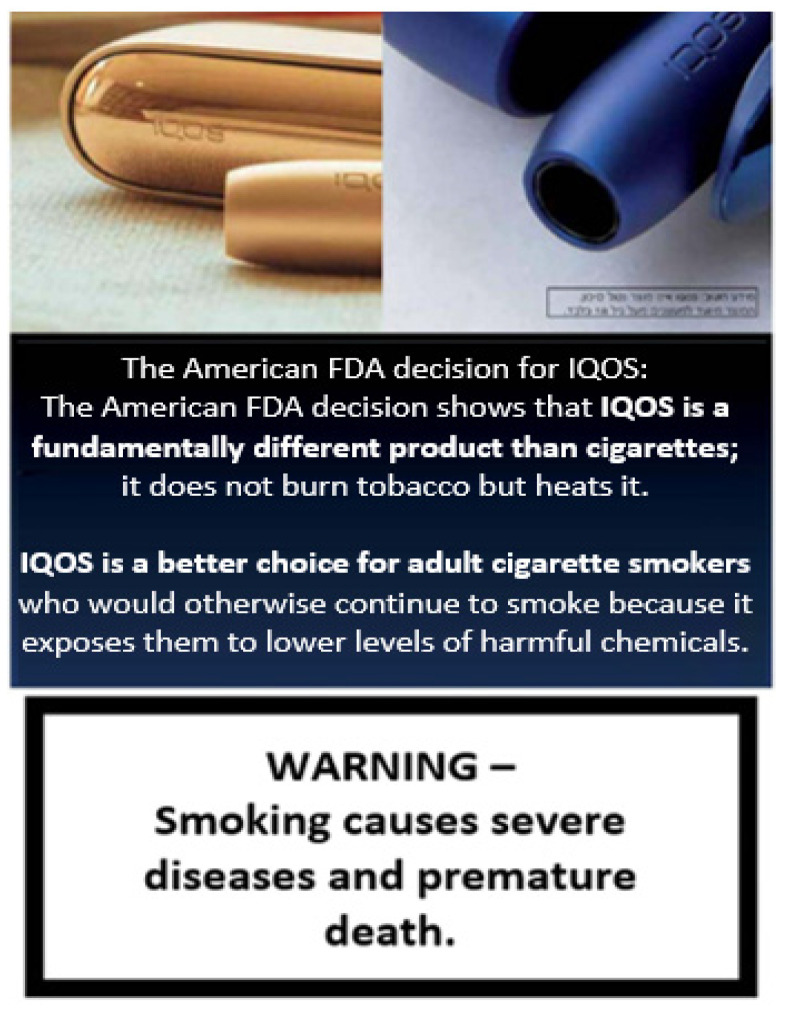
Use of health warning content and FDA MRTP in Israel IQOS ad (translated). Note: Ad from Israel in Spring 2021, purchased from Ifat group [81], initially in Hebrew and translated to English.

Notably, how consumers interpret health warning labels (HWLs) on IQOS product labeling is of concern, as PM may use anti-tobacco ads and HWLs to “position” IQOS. For example, in Israel, PM seems to be leveraging their knowledge of what mandated anti-tobacco ads were to be used each month to select the types of products and/or messages they would emphasize during that time; when anti-tobacco ads focused on smoking cessation, IQOS or other non-cigarette products were emphasized [45]. Following from this finding, one concern is that consumers may misperceive whether HWLs (i.e., “Smoking Causes Lung Cancer, Heart Disease…”, “Quitting Smoking Now Greatly Reduces Serious Risks to Your Health”) apply to the HTP or an HTP ad where the HWL is located. In fact, these warnings may be perceived as endorsements for IQOS and its advertising message, for example: “Looking for an alternative to cigarettes?” (see Figure 2 for example advertising messaging in Israel that responds to the warning). 

Another concern is PM’s exploitation of the FDA’s “reduced exposure” authorization. For example, surveillance efforts found that PM began using such language prior to MRTP authorization [82], suggesting the need to establish systems to monitor and identify industry efforts to imply reduced risk or provide other misleading information not authorized by the FDA. Furthermore, PM has used the FDA’s MRTP authorization to promote IQOS globally (see Figure 2 for example text of using FDA MRTP authorization in an Israeli ad), including efforts to minimize government regulation of IQOS [83,84,85,86,87,88,89,90,91,92,93]. Since July 2020, PM is cited in media reports in several countries mischaracterizing the FDA’s MRTP decision as evidence that IQOS is a reduced harm product [83,84,85,86,87,88,89,90,91,92,93]. Leveraging the WHO’s Framework Convention on Tobacco Control (FCTC) [94,95,96] as a basis, several countries have imposed strict regulations on IQOS marketing and sales [97] or banned its entry into their markets altogether [98,99]. Other FCTC parties have been recommended to follow [100]. While this has concerning implications for tobacco control worldwide, the fact that these types of communications are distributed online, via social media, and via other informal or less explicit ways imply potential impact among US consumers who will likely be exposed to such information.

## 5. Conclusions

In conclusion, IQOS has the potential for rapid uptake, given PM’s stated intentions to catalyze the expansion of such products [14]. This is particularly true in the US subsequent to the FDA’s MRTP authorization [5,6]. Since PM estimations of their own product’s public health impact warrant scrutiny [28,29,30,31,32,33,34,35,36], the FDA needs independent research to better inform their estimates of population impact. Unfortunately, there is insufficient research regarding key areas of concern. First, limited research [38,44,46,47,71,101,102] has examined IQOS POS, including online. Thus, more research is needed, particularly regarding PM’s innovative methods to promote IQOS at POS [37,44,45,46,47], via social media [42,52], and through other non-traditional channels or channels more difficult to systematically monitor (e.g., sponsored events, direct-to-consumer). Second, there has been limited thorough analysis of IQOS advertising content and distribution channels, which is critical for understanding target markets. Given IQOS’ potential targeting of specific subgroups (e.g., young adults) [38], which contradicts PM’s assertion that IQOS’ target market is smokers [2,67,68], this type of research is critical. Third, given the dearth of research regarding the impact of marketing allowed by the FDA’s MRTP authorization, research is gravely needed regarding the impact of health-related content in IQOS advertising [80,81,82,83,84,85,86,87,88,89,90,91,92,93]. 

It is also noteworthy that the expansion of IQOS in the newer US markets has been hampered by COVID-19 and related restrictions. Moreover, IQOS expansion is stalled currently due to a patent-infringement lawsuit against PM brought forth by British American Tobacco Plc. [103]. Once the lawsuit passes and COVID-19 becomes less relevant, one might hypothesize that IQOS marketing will quickly expand. Now is the time to prepare for the multiple strategies that will be needed to comprehensively assess IQOS’ marketing strategies. By comprehensively assessing PM’s IQOS marketing strategies as IQOS expands to new markets across the US and their impact on consumer behavior and perceptions, findings will inform estimations of IQOS’ population health impact and future FDA MRTP-related decisions, particularly as newer versions of IQOS and PM’s “reduced risk” application for IQOS are reviewed. Considering PM is a major tobacco company with a long-standing history in the US, this line of work has implications beyond IQOS and beyond PM. As prior literature has suggested, the advancement of global tobacco control efforts is contingent on translating our knowledge findings derived from research studying different tobacco companies and their products, as well as across countries with different regulatory frameworks [9,104].

## Data Availability

Not applicable.
